# Co-occurrence of bacteria and viruses and serotype distribution of *Streptococcus pneumoniae* in the nasopharynx of Tanzanian children below 2 years of age following introduction of the PCV13

**DOI:** 10.3389/fpubh.2024.1298222

**Published:** 2024-01-22

**Authors:** Matilda Emgård, Maria Andersson, Lucia Gonzales-Siles, Sia E. Msuya, Balthazar M. Nyombi, Rickard Nordén, Florida Muro, Magnus Lindh, Rune Andersson, Susann Skovbjerg

**Affiliations:** ^1^Department of Infectious Diseases, Institute of Biomedicine, Sahlgrenska Academy, University of Gothenburg, Gothenburg, Sweden; ^2^Center for Antibiotic Resistance Research (CARe), University of Gothenburg, Gothenburg, Sweden; ^3^Department of Pediatrics, Queen Silvia Children’s Hospital, Sahlgrenska University Hospital, Gothenburg, Sweden; ^4^Department of Clinical Microbiology, Sahlgrenska University Hospital, Gothenburg, Sweden; ^5^Institute of Public Health, Kilimanjaro Christian Medical University College (KCMUCo), Moshi, Tanzania; ^6^Department of Community Medicine, Kilimanjaro Christian Medical Center (KCMC), Moshi, Tanzania

**Keywords:** *Streptococcus pneumoniae*, pneumococcal conjugate vaccines, viruses, infant, sub-Saharan Africa

## Abstract

**Introduction:**

Pneumococcal conjugate vaccines have reduced severe disease attributed to vaccine-type pneumococci in children. However, the effect is dependent on serotype distribution in the population and disease development may be influenced by co-occurrence of viral and bacterial pathogens in the nasopharynx.

**Methods:**

Following introduction of the 13-valent pneumococcal conjugate vaccine (PCV13) in Tanzania we performed repeated cross-sectional surveys, including 775 children below 2 years of age attending primary healthcare centers. All children were sampled from nasopharynx and pneumococci were detected by single-target PCR. Pneumococcal serotypes/groups and presence of viruses and other bacteria were determined by two multiplex PCR assays.

**Results:**

The prevalence of PCV13 vaccine-type pneumococci decreased by 50%, but residual vaccine-types were still detected in 21% of the children 2 years after PCV13 introduction. An increase in the non-vaccine-type 15 BC was observed. Pneumococci were often co-occurring with *Haemophilus influenzae*, and detection of rhino/enterovirus was associated with higher pneumococcal load.

**Discussion:**

We conclude that presence of residual vaccine-type and emerging non-vaccine-type pneumococci in Tanzanian children demand continued pneumococcal surveillance. High co-occurrence of viral and bacterial pathogens may contribute to the disease burden and indicate the need of multiple public health interventions to improve child health in Tanzania.

## 1 Introduction

In December 2012 Tanzania implemented the 13-valent pneumococcal conjugate vaccine (PCV13), using a 3 + 0 schedule with doses given at 6, 10 and 14 weeks. PCVs have clearly reduced the burden of invasive pneumococcal disease (IPD) including meningitis in children attributed to vaccine-type (VT) pneumococci (1), and the number of childhood hospital admissions with radiology confirmed pneumonia (2). Nevertheless, *Streptococcus pneumoniae* (the pneumococcus) remains the leading cause of bacterial pneumonia in children (3) with the highest death toll in African children (4).

Colonization of pneumococci in the nasopharynx precedes disease and enables transmission (5). Currently, there are more than one hundred known serotypes of pneumococci with different capacities to cause disease or establish longer duration of carriage (6). Since surveillance of invasive disease is rare in sub-Saharan Africa, monitoring of residual VT and non-vaccine-type (NVT) pneumococci in the nasopharynx is used for studying the impact of vaccination (5). Furthermore, the nasopharynx is the primary location for interaction of co-occurring viruses and bacteria which might enhance the development of, or severity of disease (7). Recently, a large case–control study concluded that while viruses are the primary cause of most cases of childhood pneumonia requiring hospital admission in Africa and Asia, bacterial pneumonia are associated with the most severe or fatal cases (3). Meanwhile, although antibiotics are lifesaving in severe bacterial disease, recent studies reveal high inappropriate use of antibiotics in Tanzanian children (8, 9), contributing to increased antimicrobial resistance.

In the present study, we used multiplex PCR to determine pneumococcal serotypes in the nasopharynx of Tanzanian children under 2 years of age attending primary healthcare shortly after PCV13 introduction. We further aimed to explore the influence of other respiratory pathogens on pneumococcal load and parent-reported symptoms and/or antibiotic use in their children. Since local surveillance of pneumococci and other respiratory pathogens in the general child population is scarce, our results may be of importance for public health priorities in Tanzania.

## 2 Materials and methods

### 2.1 Study population

Repeated cross-sectional surveys were performed in October–November 2013 (dry or short rainy season), February–March 2014 and 2015 (warm or long rainy season) in Moshi Municipal Council, an urban area in the Kilimanjaro Region of Northern Tanzania. Recruitment of children and obtaining of sociodemographic and health information about the child, including symptoms on day of visit and antibiotic use in the past week, have been previously described (9). In short, all children below 2 years of age attending any of the appointed governmental primary healthcare facilities with their parent/guardian, and not in need of referral for hospital care, were invited to participate in the study. In total, 775 children were included, of which 173 (22%) visited the health care facility for vaccination, 389 (50%) for growth monitoring and 213 (27%) for minor illnesses or other reasons. Median age of the children was 8.7 months (8.8 in 2013, 7.7 in 2014 and 9.7 in 2015) with an approximately equal distribution among girls/boys (374/401). Vaccine uptake in the study population was high, during the study period children immunized with one or more doses of PCV13 increased from 62% (208/338) in 2013, 85% (190/224) in 2014 and 99% (210/213) in 2015.

### 2.2 Specimen collection and initial procedures at the laboratory

Each child was sampled from the nasopharynx according to standard procedures (10), using a Blue-cap E-swab (Copan Diagnostics Inc., Murrieta, United States) containing Liquid Amies medium. The samples were transported in a cool box within the same day to the Clinical Laboratory at Kilimanjaro Christian Medical Center (KCMC), Moshi, Tanzania. The nasopharyngeal samples were inoculated on goat blood agar plates for culture as previously described (9) before being frozen at −20° awaiting further transportation to the Department of Infectious Diseases, Gothenburg University, Sweden. Results regarding pneumococcal culture, antibiotic susceptibility and serotyping of the strains isolated by culture were presented previously (9).

### 2.3 Detection and serotyping of pneumococci

The nasopharyngeal samples were transported frozen to Sweden where DNA was extracted from 200 μL of the Liquid Amies medium using the MagNA Pure LC instrument (Roche Diagnostics, Mannheim, Germany) and the Total Nucleic Acid Kit (Roche Diagnostics). After the extraction, the eluted nucleic acids (100 μL) were diluted 1:2 with RNase-free water. All samples were first analyzed by a single-target polymerase chain reaction (PCR) for the “Xisco” gene, a reliable marker for pneumococcal identification (11–13). Results with a Cycle threshold (Ct) value <40 were considered positive. Positive samples were further analyzed for identification of pneumococcal serotypes or groups by a multiplex real-time PCR protocol based on sequences published by Centers for Disease Control and Prevention (CDC) and developed in-house as previously described (14). This protocol also included detection of the pneumococcal capsule *cpsA* gene. In summary, the assay could detect all VTs included in the PCV13, namely, 1, 3, 4, 5, 6ABCD, 7FA, 9AV, 14, 18, 19A, 19F and 23F, and the following NVTs 2, 7 BC/40, 8, 9NL, 10A, 11 AD, 12AF/44/46, 15 BC, 17F, 20, 22AF, 25AF/38 and 33AF/37. Since there is evidence of cross-protection with vaccine-related serotypes (15), a possible 6CD, 7A, 9A and 18 non-C was considered VTs in the analysis although not included in the PCV13.

### 2.4 Detection of other bacterial or viral respiratory pathogens

Prior to analysis of other bacterial or viral respiratory pathogens, a new extraction of nucleic acid was performed from the nasopharyngeal samples as described above. The nucleic acids were eluted in 100 μL and of these, 5 μL was used in each of eight parallel 3-plex real-time PCRs (16), together targeting 14 different viruses (adenovirus, human coronaviruses NL63, HKU1, OC43, 229E, influenza virus A and B, human metapneumovirus, parainfluenza virus 1–4, respiratory syncytial virus (RSV) and human rhinovirus/enterovirus) and four bacterial species (*Bordetella pertussis, Chlamydia pneumoniae, Haemophilus influenzae* and *Mycoplasma pneumoniae*). Results with Ct values <40 were considered positive.

### 2.5 Statistics

For the ratio between VT and all detected pneumococci, 95% confidence intervals were calculated using Agresti-Coull in IBM SPSS Statistics v. 28. Differences in the observed prevalence of VT, NVT and individual serotypes between the years and in relation to PCV13 status were examined by Fisher’s exact test in GraphPad Prism v. 9.4.1. Using GraphPad Prism the associations between pneumococcal load and presence of other respiratory pathogens (detected in >20 children) were calculated with a two-tailed, Mann–Whitney U test. Univariable and multivariable logistic regression were performed to explore associations between parent-reported symptoms or antibiotic use in the children and presence of respiratory pathogens (detected in >20 children). These analyzes were performed using IBM SPSS Statistics; the significance of coefficients was tested for using Wald’s test. *p*-values ≤ 0.05 were considered significant in all analyzes. Adjustment for multiple testing was performed by Holm’s procedure.

## 3 Results

### 3.1 Pneumococcal serotype/serogroup distribution

Pneumococci were detected in 79% (614/775) of the samples and were further analyzed for serotype or serogroup by multiplex-real time PCR. In total, 404 serotypes/groups were detected in 355 samples (307 samples with 1 serotype, 47 samples with 2 serotypes and one sample with 3 serotypes). Since the genotyping assay could identify all PCV13 VTs with high sensitivity, samples with detected pneumococci in which no serotype/group could be identified were considered as non-identified NVT pneumococci (*n* = 259). In total, a VT was detected in 30% (231/775) of the samples; but in 25 of these 231 samples, an additional NVT was present. Therefore, 53% (408/775) of all samples comprised an identified (149 samples) or non-identified (259 samples) NVT pneumococci. Notably, a fraction of the non-identified NVTs could be non-encapsulated or non-typeable pneumococci as 47 of the “Xisco” positive samples were *cpsA* negative and no serotype could be identified.

**Figure 1:**
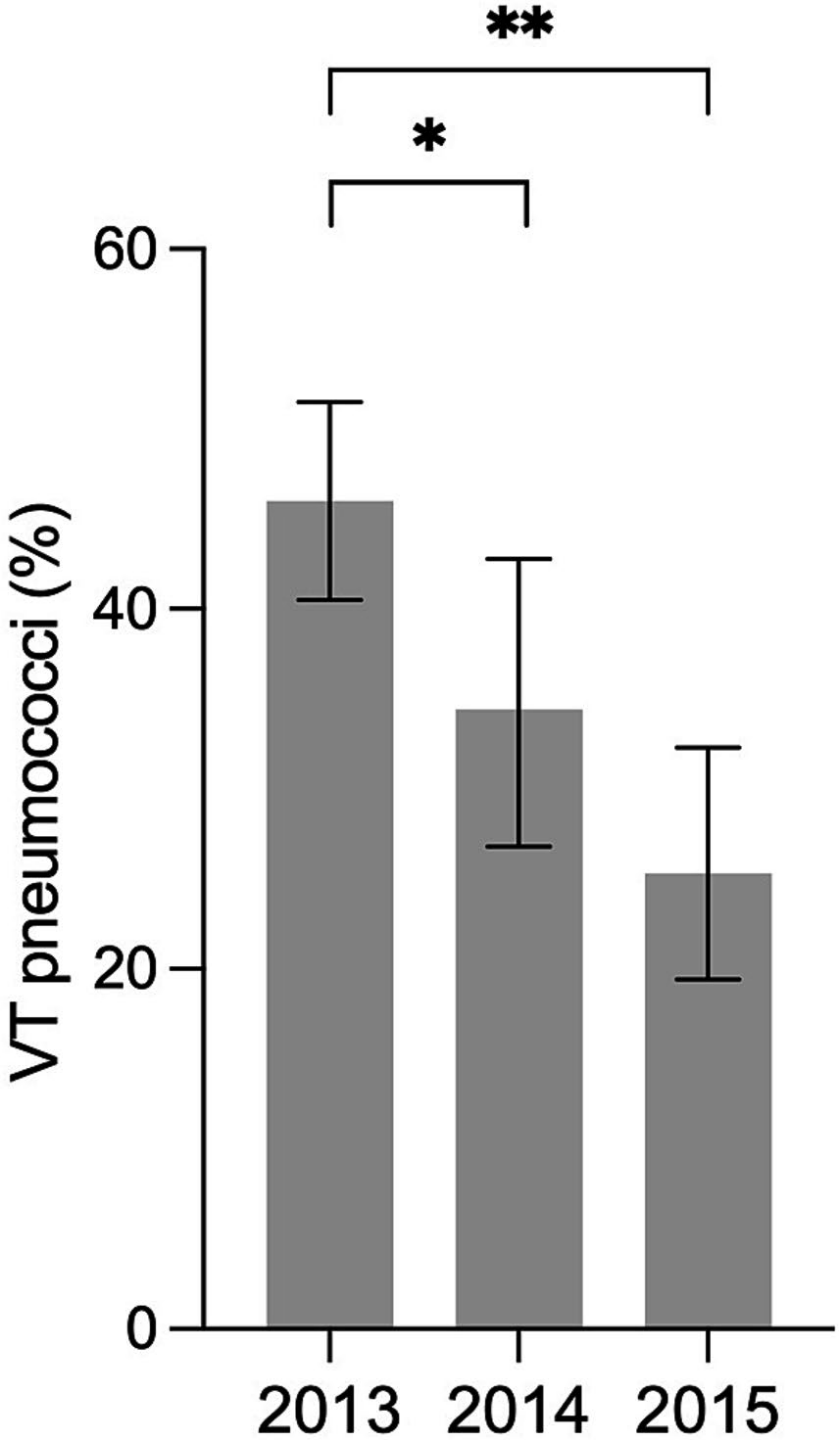
Ratio of PCV13 vaccine-type (VT) pneumococci in relation to all detected pneumococci (142/309 in 2013, 45/131 in 2014 and 44/174 in 2015) in the nasopharynx of Tanzanian children under 2 years of age. Error bars represent 95% confidence intervals; *p-value 0.027; **p-value < 0.0001.

The crude number and prevalence of detected pneumococci for each year are shown in Table 1. Crude VT prevalence decreased by half from 2013 to 2014 and 2015 (42% versus 20 and 21%, *value of p* <0.0001 for both). Between 2013 and 2015, NVTs increased, but the overall pneumococcal prevalence decreased. Adjusted for all detected pneumococci, the proportion of VTs decreased significantly from 46% in 2013, to 34% in 2014 and 25% in 2015 (Figure 1).

**Table 1:** Total number of children positive for pneumococci, PCV13 vaccine-type (VT) and non-vaccine type (NVT) (identified or non-identified).

	2013, *N* = 338	2014, *N* = 224	2015, *N* = 213	*p*-value (2013 vs. 2015)
VT, *n* (%)	142 (42)	45 (20)	44 (21)	<0.0001
NVT, *n* (%)	184 (54)	89 (40)	135 (63)	0.042
All pneumococci, *n* (%)	309 (91)	131 (59)	174 (82)	0.001

In 25 children both a VT and NVT were present.

CI; confidence interval.

**Figure 2:**
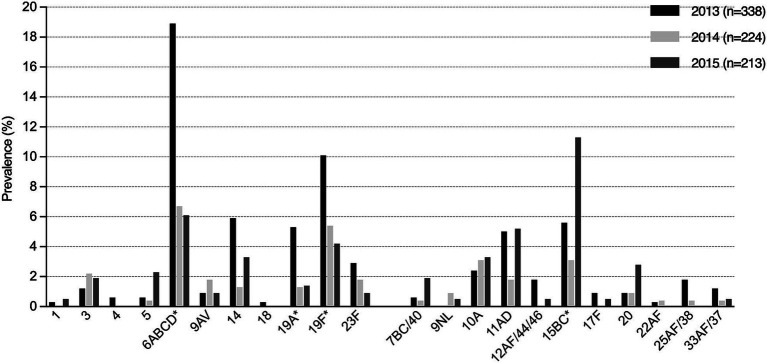
Prevalence of detected pneumococcal serotypes or groups in relation to year and inclusion in PCV13 (total number of VTs, 252 and NVTs, 152). *Between 2013 and 2015, there was a significant decrease of the detected VTs 6ABCD, 19A and 19F whilst there was a significant increase of the NVT 15BC.

The prevalence of individual serotypes or groups in relation to year and inclusion in PCV13 is shown in Figure 2. The most frequently detected was the VT serogroup 6 (*n* = 92), with a significant decrease in prevalence between 2013 and 2015 (19% vs. 6.1%, *p*-value <0.0001). The VTs 19A and 19F also decreased between 2013 and 2015 (5.3% vs. 1.4%, *p*-value 0.02 and 10% vs. 4.2%, *p*-value 0.014, respectively). Among the NVTs, 15 BC (*n* = 50) were the most common with an increase in prevalence from 2013 to 2015 (5.6% vs. 11%, *p*-value 0.02). When adjusted for multiple testing, the decrease in 6ABCD remained significant.

### 3.2 Vaccination status in relation to detection of vaccine-type pneumococci

Compared with children not vaccinated with PCV13, those with partial (1–2 doses) or full PCV13 vaccination were significantly less likely to be positive for VT pneumococci (Table 2).

**Table 2:** Detection of vaccine-type (VT) pneumococci in relation to vaccination with PCV13.

	VT *n* (%)	*p*-value^a^
No PCV vaccination, *N* = 167	75 (45)	–
Partial PCV13 vaccination, *N* = 123	35 (28)	0.0049
Full PCV13 vaccination, *N* = 485	121 (25)	<0.0001

a No PCV vaccination is used as reference category.

### 3.3 Distribution of bacterial and viral respiratory pathogens

The number and prevalence of detected respiratory pathogens other than pneumococci for each study year are shown in Table 3. Whilst detection of the bacterial species *Bordetella pertussis*, *Chlamydia pneumoniae* and *Mycoplasma pneumoniae* were uncommon, *Haemophilus influenzae* was detected in 57% (442/770) of the children. Rhino/enterovirus was the most frequently detected virus, present in almost half of the children. The prevalence of adenovirus and parainfluenza virus was similar throughout the study years whilst coronavirus was more common in 2013 (dry or short rainy season) and RSV was more common in 2015 (warm or long rainy season).

**Table 3:** Respiratory pathogens other than pneumococci detected by PCR in nasopharyngeal secretions from Tanzanian children under 2 years of age attending primary healthcare 2013–2015.

	Year			Total (*N*=770^a^) *n* (%)
	2013 (*N* = 338) *n* (%)	2014 (*N* = 219) *n* (%)	2015 (*N* = 213) *n* (%)	
*Haemophilus influenzae*	226 (67)	92 (42)	124 (58)	442 (57)
*Bordetella pertussis*	3 (0.9)	5 (2.3)	8 (3.8)	16 (2.1)
*Chlamydia pneumoniae*	2 (0.6)	1 (0.5)	0 (0)	3 (0.4)
*Mycoplasma pneumoniae*	1 (0.3)	0 (0)	0 (0)	1 (0.1)
Rhino/enterovirus	172 (51)	97 (44)	108 (51)	377 (49)
Adenovirus	40 (12)	24 (11)	23 (11)	87 (11)
Parainfluenza virus (type 1–4)	30 (8.9)	17 (7.8)	20 (9.4)	67 (8.7)
Coronavirus^b^	42 (14)	6 (2.7)	7 (3.3)	55 (7.1)
Respiratory syncytial virus	0 (0)	2 (0.9)	27 (13)	29 (3.8)
Metapneumovirus	3 (0.9)	10 (4.6)	0 (0)	13 (1.7)
Influenza A virus	2 (0.6)	0 (0)	0 (0)	2 (0.3)
Any virus^c^	233 (69)	134 (61)	147 (69)	514 (67)

a A new extraction of nucleic acid for the analysis was not possible in 5 of the 775 nasopharyngeal samples.

b Type NL63, HKU1, OC43, 229E.

c In 104 children 2 viruses were detected and in 6 children 3 viruses were detected.

### 3.4 Co-occurrence of respiratory pathogens in relation to pneumococcal detection

**Figure 3:**
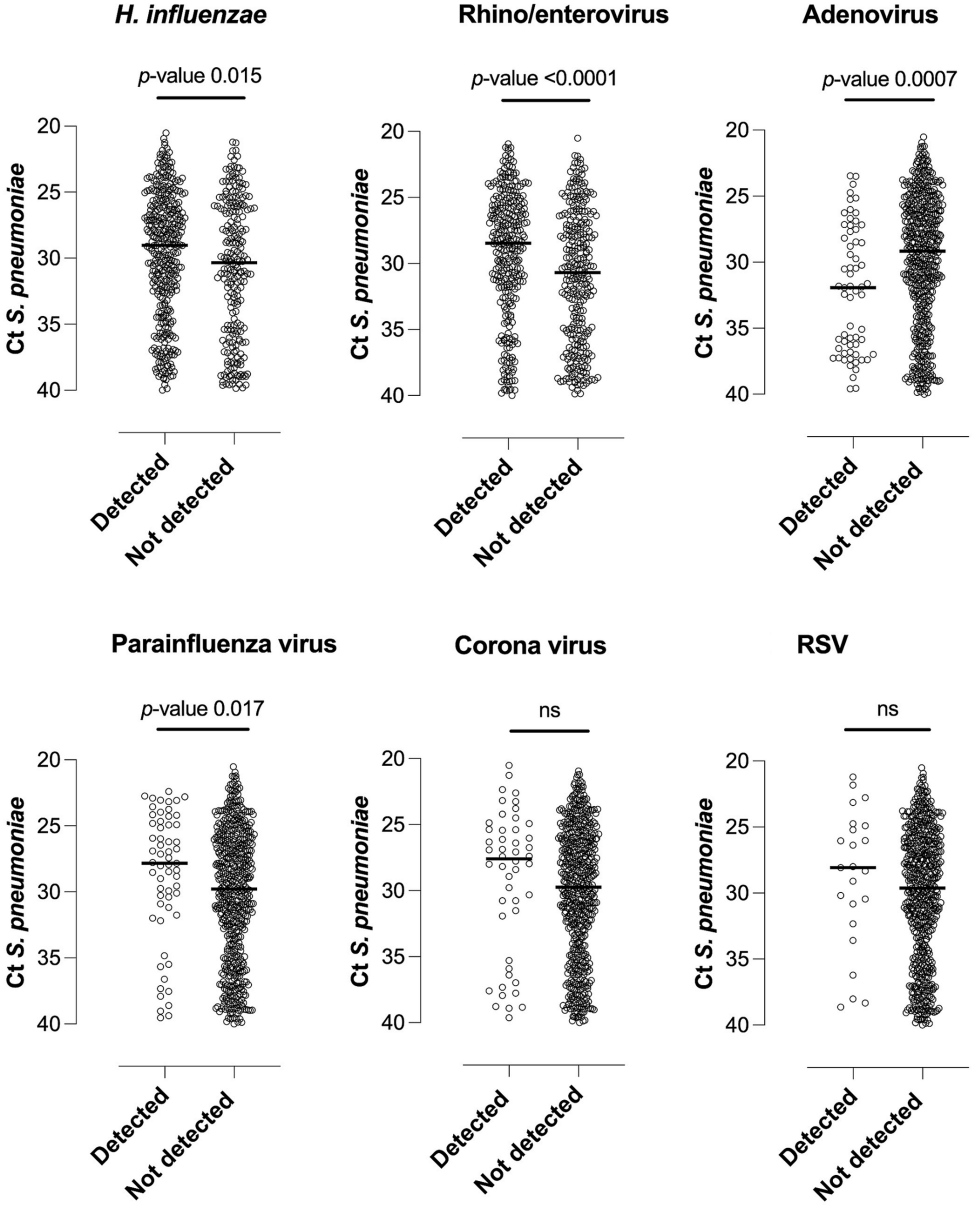
Pneumococcal load in relation to detection of other potential respiratory pathogens. The Ct (cycle threshold) value is inversely proportional to the amount of nucleic acid; lower Ct values thus relate to higher pneumococcal load. RSV, respiratory syncytial virus; ns, non-significant.

Co-occurrence of pneumococci and different viruses or other bacteria was common (Table 3). Adjusted for presence of other commonly detected respiratory pathogens, children with pneumococci were significantly more likely to also be positive for *H. influenzae* (aOR 2.53, 95% CI 1.73–3.69, *p*-value <0.001) but less likely to be positive for adenovirus (aOR 0.48, 95% CI 0.28–0.83, *p*-value 0.009) (Supplementary Table S1).Detection of *H. influenzae*, rhino/enterovirus and parainfluenza virus were associated with higher load of pneumococci (lower Ct value) and adenovirus with a lower load of pneumococci (Figure 3). Detection of coronavirus or RSV showed no significant correlation with pneumococcal load. When adjusted for multiple testing rhino/enterovirus remained indicative for a higher pneumococcal load and adenovirus for a lower pneumococcal load.

### 3.5 Detection of respiratory pathogens in relation to parent-reported symptoms and antibiotic use

Multivariable analysis was performed to assess parent-reported symptoms and antibiotic use in relation to presence of pneumococci or any other potential pathogen (Supplementary Table S2). Presence of pneumococci was weakly related to cough (aOR 1.58, 95% CI 1.03–2.42, *value of p* 0.037). Cough was also associated to parainfluenza virus, corona virus and RSV. Parent-reported fever was correlated to detection of adenovirus, parainfluenza virus and RSV. Runny nose was associated with all viruses included in the multivariable model except adenovirus, but also with detection of *H. influenzae* (aOR 1.44, 95% CI 1.05–1.98, *p*-value 0.024). Both RSV and adenovirus was associated with parent-reported rapid or difficult breathing (aOR 9.36, 95% CI 3.65–23.97, *p*-value <0.001 and aOR 2.45, 95% CI 1.08–5.56, *p*-value 0.032, respectively) and antibiotic use in the past week (aOR 4.15, 95% CI 1.92–8.95, *p*-value <0.001 and aOR 1.69, 95% CI 1.00–2.84, *p*-value 0.048, respectively).

## 4 Discussion

Between 2013 and 2015, following introduction of the PCV13 in late 2012, we observed a significant decrease of VT pneumococci in nasopharyngeal samples from Tanzanian children under 2 years of age, probably reflecting a protective effect of the vaccine. The finding that children with partial or full vaccination significantly less often carried a VT pneumococcus also support protection by the PCV13. Although the prevalence of VTs during the study period decreased by half, residual prevalence of VT pneumococci remained high (21%). This is in line with studies from The Gambia (17) and Malawi (18) three to seven years post-PCV13 introduction, also using a 3 + 0 schedule. Studies from high-income countries shows lower residual carriage of VTs in pre-school children, largely reflecting lower rates of overall pneumococcal carriage and transmission (18). Nevertheless, as reported for Europe (19) invasive pneumococcal disease (IPD) was reduced by at least 50% among pre-school children three to 7 years following PCV13 implementation in The Gambia (20) and Malawi (1). Meanwhile, NVT disease have remained low despite serotype replacement in carriage (17, 18). Considering this, vaccination with PCV13 is expected to have a substantial effect on child mortality due to IPD in Tanzania. Recently, concerns have been raised as to whether the 3 + 0 dosing schedule provides sufficient long-term protection in countries with high disease burden (21). Ongoing studies directly comparing the 3 + 0 vs. 2 + 1 schedules (with two primary doses given at 6 and 14 weeks and a booster at 9 months) will provide information as to whether adjustments may improve herd immunity, and consequently the protection of neonatal children too young to have received PCV13 vaccination (22).

Compared with other PCV13 serotypes, the effectiveness in protection against carriage and disease by serotype 3 has been poor (23). Indeed, in Europe there has been an increase in IPD in recent years, partly due to serotype 3 (19). In our study, serotype 3 was detected in 1–2% of the children, numbers were too few to reveal any change in prevalence. However, we observed a decrease in VT 6ABCD, 19A and 19F, which were also the most prevalent serotypes/groups in Zanzibar before introduction of the PCV13 (24). Similarly, in Kilifi, Kenya, a significant decrease in 6B and 19F was reported in children <5 years 2 years after PCV10 introduction (25) but remaining residual carriage of 19F has also been observed in South Africa (26) and The Gambia (17). Importantly, we observed an increase in the NVT 15 BC, the most common cause of meningitis in children under-five in countries using the PCV10/13 (21). In South Africa, 15 BC has also been recognized an important cause of IPD (27) and was among the most common NVT detected in healthy children post-PCV13 (26). In conclusion, the long-term effects of PCV13 use in both high-and low-income countries are promising, but continued surveillance of the impact of emerging NVTs and residual VTs impact on pneumococcal carriage and disease in children is warranted, especially in areas with high transmission (21).

It must be noted that the effect of PCV on nasopharyngeal carriage of pneumococci has mostly been studied by culture-based methods, whilst here we used molecular-based methods. To illustrate, cultures performed on the nasopharyngeal samples analyzed in this study could only detect pneumococci in 31% of the children (9) compared to 79% by PCR (as presented above). The higher sensitivity of the molecular methods, and the fact that a possible 6CD, 7A, 9A and 18-nonC can be misclassified as a PCV13 VT, may contribute to the slightly higher VT prevalence seen in the present study.

This study reveals high occurrence of both respiratory viruses and bacteria in children attending primary healthcare in Northern Tanzania, the large majority for growth monitoring or vaccination. Rhinovirus or enterovirus were detected in almost half of the children in our study. The method did not distinguish between these viruses, but the large majority likely were rhinovirus (28). Detection of rhino/enterovirus showed a significant association with higher load of pneumococci, similar to observations of under-five children in the United Kingdom (29). Furthermore, higher pneumococcal load in nasopharynx has been associated with microbiologically confirmed pneumococcal pneumonia in children (30), indicating that although rhinovirus is a common and well-known cause of upper respiratory tract disease, it may increase the likelihood of severe pneumococcal disease.

We observed a possible epidemic of RSV in 2015 when sampling took place during the warm or long rainy season. Although children in 2014 were recruited during the same period, few children were positive for RSV, and during the dry or short rainy season 2013 RSV was not detected in any case. In tempered countries, RSV typically causes biannual epidemics during the cold, winter months, although restrictions due to the COVID-19 pandemic recently disrupted this pattern. It was recently concluded that RSV indeed has clear seasonal epidemics in tropical regions as well, in the southern hemisphere correlating to the first half of the year (31). Although several studies suggest a bidirectional interaction between both RSV and influenza virus and pneumococci in the nasopharynx which increases the likelihood of disease (7, 32), this study could neither show any association between detection of pneumococci in presence of RSV or influenza virus probably due to the few cases of these viruses. Meanwhile presence of the more prevalent parainfluenza virus was associated with higher pneumococcal load, and parainfluenza has been associated with pneumococcal acquisition in a case–control study involving children <3 years in Peru (33).

Importantly, co-occurrence of pneumococci and *H. influenzae* in the children was shown across several analyzes. Non-typeable *H. influenzae* and pneumococci are known to form multispecies biofilms in the respiratory tract (34). A case–control study from Moshi indicated an association between co-occurrence of these pathogens with community acquired pneumonia in children under-five (34) with most isolated *H. influenzae* being non-typeable (96%). In the present study, no virus was found to be more frequently co-occurring with pneumococci, but adenovirus was less likely to co-occur with and was correlated with a lower pneumococcal load. This is in line with the previously mentioned study in Peru (33), where infection with adenovirus did not favor pneumococcal acquisition.

Only two parent-reported symptoms were associated with detection of bacteria, namely cough, associate to presence of pneumococci and runny nose to detection of *H. influenzae*, although these symptoms were also strongly correlated to various respiratory viruses. Interestingly, parent-reported fever, cough, rapid/difficult breathing, and antibiotic use in the past week were associated with detection of RSV, known to be the most common etiology of bronchiolitis or pneumonia in children (3). Rapid/difficult breathing and antibiotic use were also associated with detection of adenovirus. Although adenovirus may cause severe pneumonia, its etiological share in childhood pneumonia is less known (3). Cough and rapid/difficult breathing are the main clinical symptoms indicating pneumonia according to the Integrated Management of Childhood Illness; the recommended treatment is oral amoxicillin in non-severe cases. In this study, over half (54%) of the children had received antibiotics in the past 3 months and almost 1/5 in the past week, with amoxicillin being the most widely used antibiotic (9). Although these high numbers suggest broad inappropriate use, our data indicate that viruses which generally cause more severe symptoms in children were more likely to be treated with antibiotics. In a setting with few diagnostic tools and high childhood mortality, this may to some extent be argued as non-irrational use. In addition, co-occurrence of virus and bacteria in the nasopharynx includes both synergistic and antagonistic effects (32), further influenced by the host immune defense. This highlights the complexity in the etiology of respiratory tract disease in children and the importance of underlying factors affecting the susceptibility or severity of disease, such as malnutrition (3). In relation to need for antibiotics, a terminology focusing on whether the infection in the individual child could be self-limiting with symptomatic, but not antibiotic treatment, or whether the infection in the individual child does indeed require antibiotics, may be more beneficial than terminology solely focusing on the distinction between viral and bacterial cause.

This study has several limitations. Sampling took place during dry or short rainy season in 2013 and warm or long rainy season in 2014–2015. Pneumococcal carriage prevalence may differ in relation to season, both rain or cold weather may incite people to spending more time indoors, thus contributing to crowding and thereby pathogen transmission. Furthermore, different clinics were included in 2014 compared with 2013 and 2015, although these areas had similar socioeconomic standard. In 2014 we observed lower overall prevalence of pneumococci. Similar trends have been observed in Kilifi, Kenya where NVT pneumococci decreased at an early stage of vaccine implementation with a subsequent increase (25). It is likely that NVT pneumococci may have increased further after the study period, which will also affect the overall pneumococcal prevalence. Although secular changes may affect the results of this exploratory study, we suggest our observed decrease of VT pneumococci results from a direct effect of the PCV13, rather than seasonal variations or differences in locality. Lastly, symptoms and antibiotic use in the children were largely dependent on parental recall which were not verified by the researchers, consequently, the true use of antibiotics may be even higher than reported here (35).

## 5 Conclusion

Shortly following introduction of the PCV13 in Tanzania, VT pneumococci detected in the nasopharynx of children under 2 years was reduced by around 50%. Thus, the impact of the PCV13 on severe pneumococcal disease in Tanzanian children are expected to be substantial. In line with other sub-Saharan Africa studies, residual VT pneumococci remained high and may reduce indirect effects of the vaccine. We further observed an increase in NVT 15 BC which may be of clinical importance and highlights the need for continued surveillance in the post-PCV13 era. High co-occurrence of viral and bacterial pathogens in the nasopharynx of the children attending routine healthcare may contribute to development of disease. It also indicates that multiple public health interventions are warranted to improve child morbidity and mortality in low-income countries.

## Data availability statement

The raw data supporting the conclusions of this article will be made available by the authors, without undue reservation.

## Ethics statement

The studies involving humans were approved by Kilimanjaro Christian Medical University College Research Ethics and Review Committee in Moshi, Tanzania (No. 661 and 809), the National Institute for Medical Research in Dar es Salaam, Tanzania (Vol. IX/2363) and the Regional Ethics Committee in Gothenburg (413-15). The studies were conducted in accordance with the local legislation and institutional requirements. Written informed consent for participation in this study was provided by the participants’ legal guardians/next of kin.

## Author contributions

ME: Formal analysis, Investigation, Writing – original draft, Writing – review & editing. MA: Investigation, Writing – review & editing. LG-S: Investigation, Writing – review & editing. SM: Conceptualization. BN: Conceptualization, Writing – review & editing. RN: Investigation, Writing – review & editing. FM: Writing – review & editing. ML: Formal analysis, Writing – review & editing. RA: Conceptualization, Writing – review & editing. SS: Conceptualization, Funding acquisition, Writing – review & editing.
