# Supplementary material for: Co-occurrence of bacteria and viruses and serotype distribution of Streptococcus pneumoniae in the nasopharynx of Tanzanian children below 2 years of age following introduction of the PCV13
Source: Front Public Health. 2024 Jan 22;12:1298222. doi: 10.3389/fpubh.2024.1298222 (PMC10839969; doi:10.3389/fpubh.2024.1298222)
Supplement: Supplementary file 1 [file Data_Sheet_1.docx]

Supplementary Material

**Supplementary Table 1.** Detection of *S. pneumoniae* (79%, 610/770) in relation to co-occurrence of other respiratory pathogens.

|  |  | Univariable analysis | | | Multivariable analysis^a^ | | |
| --- | --- | --- | --- | --- | --- | --- | --- |
|  | Co-occurrence  *n/N* (%) | OR | CI (95%) | *p*-Value | OR | CI (95%) | *p*-Value |
| *Haemophilus influenzae* | 382/442 (86) | 2.79 | 1.95-4.00 | <0.001 | **2.53** | **1.73-3.69** | **<0.001** |
| Rhino/enterovirus | 311/377 (82) | 1.48 | 1.04-2.11 | 0.029 | 1.36 | 0.94-1.97 | *NS* |
| Adenovirus | 63/87 (72) | 0.65 | 0.39-1.08 | *NS* | **0.48** | **0.28-0.83** | **0.009** |
| Parainfluenza virus (type 1-4) | 59/67 (88) | 2.03 | 0.95-4.35 | *NS* | 2.18 | 0.99-4.80 | *NS* |
| Coronavirus | 48/55 (87) | 1.87 | 0.83-4.21 | *NS* | 1.91 | 0.83-4.42 | *NS* |
| Respiratory syncytial virus | 23/29 (79) | 1.01 | 0.40-2.51 | *NS* | 0.86 | 0.33-2.26 | *NS* |

^a^Adjusted for age and respiratory pathogens included in the univariable analysis.

**Supplementary Table 2.** Multivariable analysis of associations between detected pathogens and symptoms or antibiotic use as reported by parents/guardians in 770 Tanzanian children under two years of age attending primary healthcare.

|  | Fever   118 (15 %) | Runny nose  340 (44 %) | Cough  279 (36 %) | Rapid or difficult breathing 41 (5 %) | Antibiotic use  last week 150 (19 %) |
| --- | --- | --- | --- | --- | --- |
|  | aOR^a^ (95 % CI) | aOR^a^ (95 % CI) | aOR^a^(95 % CI) | aOR^a^ (95 % CI) | aOR^a^ (95 % CI) |
| *S. pneumoniae* | 1.11 (0.64–1.92) | 0.95 (0.65-1.41) | **1.58 (1.03-2.42)*** | 0.63 (0.28-1.40) | 0.65 (0.42-1.02) |
| *H. influenzae* | 1.38 (0.89-2.14) | **1.44 (1.05-1.98)*** | 1.36 (0.98–1.89) | 1.81 (0.87–3.78) | 1.04 (0.71-1.53) |
| Rhino/enterovirus | 0.87 (0.58-1.32) | **2.41 (1.77-3.28)***** | 1.36 (0.99–1.86) | 0.99 (0.51-1.92) | 1.11 (0.77–1.61) |
| Adenovirus | **1.88 (1.07-3.32)*** | 0.76 (0.46-1.23) | 0.87 (0.53-1.44) | **2.45 (1.08-5.56)*** | **1.69 (1.00-2.84)*** |
| PIV^b^ (type 1-4) | **4.25 (2.42-7.44)***** | **3.84 (2.18-6.76)***** | **5.7 (3.21-10.13)***** | 2.53 (0.98-6.54) | 0.97 (0.50-1.89) |
| Coronavirus | 1.10 (0.49-2.45) | **1.87 (1.05-3.23)*** | **1.96 (1.11-3.48)*** | 1.42 (0.41-4.94) | 0.95 (0.45-2.02) |
| RSV^c^ | **2.86 (1.23-6.64)*** | **3.28 (1.46-7.41)**** | **3.05 (1.39-6.69)**** | **9.36 (3.65–23.97)***** | **4.15 (1.92–8.95)***** |

CI; confidence interval

* *p*-value <0.05

** *p*-value <0.01

*** *p*-value <0.001

^a^Adjusted for age and the other pathogens listed in the table.

^b^Parainfluenza virus

^c^Respiratory syncytial virus
